# Identifying and Addressing Distress in Cancer Care: A Study of Screening Outcomes and Referrals in Australian Oncology Outpatients

**DOI:** 10.1002/pon.70584

**Published:** 2026-08-20

**Authors:** E. Matthews, K. Webber, C. Parker, C. Feola, T. Gencarelli, P. Koe‐Leong, J. F. Wiley

**Affiliations:** ^1^ School of Psychological Sciences Faculty of Medicine, Nursing and Health Sciences Monash University Clayton Victoria Australia; ^2^ Oncology Department Monash Health Clayton Victoria Australia; ^3^ School of Clinical Sciences Monash University Clayton Victoria Australia; ^4^ School of Public Health and Preventive Medicine Monash University Clayton Victoria Australia; ^5^ Supportive and Palliative Care Unit Monash Health Clayton Victoria Australia

**Keywords:** cancer, distress, health resources, oncology, psycho‐oncology

## Abstract

**Objective:**

Psychological distress is highly prevalent among oncology patients and represents an important unmet supportive care need. Yet referrals made following routine distress screening remain poorly understood. This study examined referrals following routine distress screening in outpatient oncology to assess how screening supports patient care, especially for psychological concerns.

**Methods:**

All outpatient medical oncology distress screenings at a major metropolitan Victorian hospital from 1 December 2023 to 31 May 2024 were extracted from medical records. Screening was conducted with the NCCN distress thermometer (DT), problem list and additional custom items. Patient characteristics, screening uptake, and referral following screening were analysed.

**Results:**

Of 850 distress screening encounters offered to 773 patients, 82 (9.6%) were declined, leaving 768 completed screening encounters. Among respondents, 53.6% reported clinically significant distress (DT ≥ 4) and 60.4% reported psychological concerns. 35.8% (*n* = 275) of screenings resulted in at least one referral. Patients with more distress and greater patient‐reported need for support were more likely to be referred (both *p* < 0.001). However, only a small proportion of referrals following screening targeted psychological concerns directly, with just five directed to specialist mental health services.

**Conclusions:**

Distress and psychological concerns are highly prevalent in outpatient oncology, yet referral following screening often does not align with concerns reported. Most referrals are made to external services, limiting integration with oncology care. Improving referral pathways and enhancing access to mental health services within oncology settings are critical steps towards better meeting patients' psychological needs and ensuring that distress screening translates into meaningful, patient‐centred care.

## 1 Background

Distress during cancer is defined as a “multifactorial, unpleasant experience of a psychologic (i.e., cognitive, behavioural, emotional), social, spiritual, and/or physical nature that may interfere with the ability to cope effectively with cancer, its physical symptoms, and its treatment” [1]. While guidelines recommend routine distress screening, there is limited evidence about how screening results drive referral following screening, and how these referrals align with patient‐reported need for support.

Sources of distress during treatment for cancer are varied [2], with psychological distress conferring a disproportionate influence on the overall distress levels of people being treated for cancer [3, 4]. Patients experiencing psychological distress present more frequently for care [5, 6], are at a greater risk for hospitalization and longer inpatient stays [7] and have poorer disease‐related outcomes [8].

Despite recognition of psychological distress management as an unmet need in oncology [9, 10], implementation of distress screening results to guide clinical care and referral to support services appears inconsistent [11]. Screening is intended to facilitate timely connection to appropriate supportive care [12, 13], and screening alone does not translate into improved patient outcomes [14, 15]. Australian cancer survivors do not access mental health supports at a higher rate than the general population [16] suggesting that translation of screening into service utilisation may be inefficient.

In Australia, supportive care for cancer patients is primarily delivered through the hospital system, though access and service delivery vary significantly between institutions. Some centres have specialised psycho‐oncology services, while other supportive care needs may be addressed by social work [17]. External services, including telephone‐based and community services [17, 18] may provide additional options for support. However, access to community services may be limited by geographical factors, out‐of‐pocket costs and may lack cancer‐specific expertise [19]. These service variations highlight the importance of understanding how distress screening results are used to guide referral and access to psychological support within routine oncology care.

The lack of research around how and when distress screening results are being utilised to connect patients with services is a limitation. Understanding how distress screening results are being operationalised in practice may have implications for resource allocation where referral after screening does not reflect common concerns expressed by patients. Improved understanding of how distress screening results are translated into care could assist with ensuring funders and institutions are prioritising services that will be most relevant to the needs of their patients.

This study aimed to characterise a population of oncology patients who underwent distress screening within routine clinical care (including levels of psychological distress and patient‐reported need for support) and to examine any differences in uptake of distress screening. Additional aims were to describe referrals following screening, and factors associated with likelihood of referral following screening, particularly for psychological distress.

## 2 Methods

A retrospective, observational cohort study design was used to characterise distress and referral following screening amongst medical oncology outpatients screened by nursing staff over a 6‐month period at a major Victorian metropolitan health service. This study follows the STROBE guidelines for reporting observational research [20]. Monash Health Human Research Ethics Committee reviewed and approved this project (Approval number: RES‐24‐0000‐101Q). In accordance with local ethical guidelines and institutional policy, individual informed consent was not required given the use of pre‐existing data that was collected as part of routine clinical care.

### 2.1 Study Population

Data came from 850 distress screening records extracted from medical records of 773 oncology outpatients. Eligibility criteria included the completion of the Oncology Supportive Care Screening Tool (OSCST) between 1 December 2023 and 31 May 2024. Exclusion criteria included patients with a primary diagnosis of haematological malignancy, patients who were admitted at time of screening, and individuals under the age of 18.

### 2.2 Data Collection

Eligible medical records were identified by the research team using an internal search function to identify patients with OSCST administered during the study window. The OSCST was administered by nursing staff as part of usual clinical practice, in either the chemotherapy day unit or during outpatient clinic appointments. Screening encounters recorded in medical records reflected routine clinical practice within this setting. The timing of distress screening was determined by clinical workflow and judgement rather than by a structured screening schedule as per local policy. Referrals made following screening were also actioned by nursing staff.

Demographic and clinical data including age, sex, primary diagnosis, treatment intent and patient's primary language spoken were extracted for the study population.

Remaining data was collected from the hospital's OSCST which is completed as a paper form before being scanned to the patient's medical record. Variables extracted from the OSCST for this study are listed below. The Distress Thermometer (DT) and problem list [1] is a tool used to evaluate distress levels amongst patients with cancer. The distress thermometer utilises an 11‐point Likert scale visual thermometer, ranging from 0 (no distress) to 10 (extreme distress) to ask patients the level of distress they have experienced in the last week. Higher scores (DT score ≥ 4) indicate clinically significant distress [21]. The scale has been validated for use in oncology populations [22] and has demonstrated strong cross‐cultural validity [23]. The problem list includes 43 items associated with 5 symptom domains (practical, emotional, spiritual or religious, social and physical). Data from all domains of the problem list were extracted from the screening tool. In this study, endorsement of items within the emotional domain of the problem list was used as an indicator of psychological distress. Patient reported need for support was based on the question “How much help do you need with these concerns?” which was measured on an 11‐point visual thermometer, ranging from 0 (no help) to 10 (extreme help). This item is described as patient‐reported need for support throughout this text. Referrals made as a result of distress screening were documented in an open‐text field within the screening tool. Referral destinations, which were typically recorded as a few words (e.g., “social work”) or a common hospital abbreviation (e.g., “SW” for social work) were reviewed by the research team and grouped into predefined referral categories based on clinical referral pathways. Where handwritten notes were unclear, the original record was reviewed by two members of the research team and classified by consensus.

Where patients were documented to have been offered distress screening but declined, an incomplete OSCST was uploaded to the medical record as evidence of the encounter. As such, demographic and diagnostic data to describe those patients who were not screened for distress were available for extraction. For patients who had more than one distress screening encounter during the review period, each individual distress screening was included in the analysis.

### 2.3 Data Analysis

Data were extracted and cross‐checked by at least two independent researchers to ensure accuracy and uniformity. Data were cleaned using MS Excel before being analysed using R (V4.4.1). Descriptive statistics and percentages were used to summarise characteristics of the study population, distress levels and problem list endorsements, and referrals following screening. All screenings were analysed individually, regardless of whether a patient completed more than one during the eligibility period. Demographic data reflects the total sample of completed screenings. Generalized estimating equation (GEE) [24] logistic regression was used to account for the small number of patients with repeated distress screenings in the dataset. GEE models were applied both to compare referrals following screening between patient groups and to examine associations between patient‐reported need for help, DT scores, and the likelihood of referral following screening. Results are presented as average marginal effects on the predicted probability of referral.

## 3 Results

A total of 850 distress screening encounters were offered to 773 individuals during the eligibility period. Of the 850 distress screening encounters offered, 768 were completed and 82 were declined. 73 patients were offered distress screening twice within the study period, two patients were offered distress screening three times. All distress screening encounters were administered by nursing staff.

### 3.1 Population Characteristics

Characteristics of the study population who completed distress screening are presented in Table 1. The study population had a mean age of 63.3 years (SD 13.0) with 53.6% of the study population reporting clinically significant distress (distress thermometer score ≥ 4). Characteristics of the study population who declined distress screening are provided in Supporting Information S1: Table 1. Patients under 65 years were more likely to decline distress screening than patients 65 and over (*p* < 0.001). Patients whose primary language was not English were also more likely to decline distress screening compared to English speaking patients (*p* < 0.001). Differences in screening completion were observed across tumour stream groups (*p* < 0.001) and treatment intent categories (*p* < 0.001).

**TABLE 1:** Characteristics of the study population who completed distress screening.

Age, *N* (%)	
Adults (age < 65 years)	390 (50.8)
Older adults (age ≥ 65 years)	378 (49.2)
Language, *N* (%)	
English	598 (77.9)
Language other than English	170 (22.1)
Sex, *N* (%)	
Female	423 (55.1)
Male	345 (44.9)
Tumour stream, *N* (%)	
Brain	10 (1.3)
Breast	152 (19.8)
Genitourinary	73 (9.5)
Gynaecological	81 (10.6)
Head and neck	83 (10.8)
Lower gastrointestinal	122 (15.9)
Lung	124 (16.1)
Skin	18 (2.3)
Unknown primary	5 (0.7)
Upper gastrointestinal	100 (13.0)
Treatment intent, *N* (%)	
Curative	406 (52.9)
Palliative	310 (40.4)
Undetermined^a^	52 (6.8)

^a^ Treatment intent was classified as undetermined when treatment planning had not been communicated to patient at time of screening.

Endorsement rates of each domain of the problem list are shown in Table 2. All individual problem list items and their endorsement rates are shown in Supporting Information S1: Table 2. Physical and emotional concerns were the most frequently endorsed problem list domains. Within the emotional concern domain, the most commonly reported items were worry or anxiety (47.3%), followed by sadness or depression (24.8%) and fear (21.4%).

**TABLE 2:** Distress screening endorsement across completed screens.

	*N* (%)
Physical concerns	495 (64.5)
Emotional concerns	464 (60.4)
Practical concerns	336 (43.8)
Social concerns	135 (17.6)
Spiritual or religious concerns	71 (9.2)

*Note:* All individual problem list items and number of screens endorsed are shown in Supporting Information S1: Table 2.

#### 3.1.1 Referrals Following Screening

Overall, 64.2% of screenings resulted in no referrals, 24.5% resulted in one referral, and 11.3% resulted in two or more referrals (detailed in Supporting Information S1: Table 3). Among screening records that endorsed a clinically significant level of distress (DT ≥ 4) 55.6% received no referral following screening.

Referral rates differed by language and sex. Non‐English speakers were referred more frequently than English speakers (43.5% vs. 33.6%, respectively, *p* = 0.017) and females were referred at a lower rate than males (32.6% vs. 39.7%, respectively; *p* = 0.041). There was no significant difference in referrals following screening by age groups (*p* = 0.58). Destination of referrals made following screening are in Table 3. Referrals are broken down by demographic groups in Supporting Information S1: Table 4.

**TABLE 3:** Referral destinations following screening.

Service	Number of referrals made to this service	% of overall referrals
Cancer council Victoria	163	41.4
Physical support^a^	119	30.2
Social work	39	9.9
Palliative care	22	5.6
Cancer support nurses	22	5.6
Practical support^a^	16	4.1
Cancer‐specific services^a^	8	2.0
Psycho‐oncology	5	1.3

^a^ Referrals have been categorised as follows: physical support referrals (dietician, dentist, pain service, diabetes nurses, rehab, physiotherapy, Quit line), practical support referrals (My Aged Care, transport services, refugee services and Department of Veterans Affairs), and cancer‐specific services (Lung Foundation, PanCare, Look Good Feel Better).

### 3.2 Factors Associated With Likelihood of Referral

Association between endorsement of emotional concerns and clinical distress (DT ≥ 4) with referral following screening was examined. A significant association was found for referrals to the Cancer Council (*p* < 0.01). No significant associations were observed between emotional concerns, distress, and referral to Social Work (*p* = 0.13). Due to the small number of referrals to psycho‐oncology (*n* = 5), analysis of factors associated with referral could not be reliably undertaken.

Logistic regression models estimated the average marginal effect of patient‐reported need for help and distress on any referral likelihood. A one‐unit increase in patient‐reported need for help was associated with a 3.8% point increase in the probability of referral (*p* < 0.001). A one‐point increase in DT score was associated with a 2.9% point increase in the likelihood of referral (*p* < 0.001).

## 4 Discussion

Psychological distress was highly prevalent among patients who underwent distress screening, consistent with previous research [3, 9, 10, 25]. There were differences in uptake of distress screening between different demographic groups. This may reflect differences in help‐seeking behaviour due to societal norms [26], as well as patient perceptions of the purpose of screening [27], or preferences for self‐management [28]. Non‐English speakers were more likely to decline screening than English speaking patients. Although reasons for declining screening were not captured in this study, this finding may reflect potential barriers related to delivery of screening to culturally and linguistically diverse populations. Interpreter services are not guaranteed to be available at the time of screening, which may limit accessibility of screening and patients ability to communicate concerns. The use of family members in the absence of interpreter services may also influence willingness to participate in screening among non‐English speaking patients. While the available data do not allow definitive conclusions regarding the mechanisms underlying observed differences, findings highlight the importance of considering accessibility and cultural factors when implementing screening. Variation in screening uptake may influence which patients are identified as requiring support and who are subsequently connected with available care.

Higher distress levels and patient‐reported need for support were associated with an increased likelihood of referral following screening, although alignment between referrals and patients expressed concerns were not always clear, especially regarding psychological distress. Most referrals were directed to services external to the hospital, rather than being integrated within the oncology care team, and only a small number of referrals went to hospital‐based psycho‐oncology services. This pattern may reflect that in the absence of appropriate internal services, staff report referring patients to the options that are most readily available [29], even when these do not fully align with patients' reported needs. This suggests that referral patterns following screening may reflect not only patient need, but also the structure and availability of supportive care services within oncology. This finding also reflects known limitations related to availability of appropriate staff such as psychologists when referring to psychosocial care in oncology [11, 27] and suggests that reduced use of mental health services previously seen in Australian cancer survivors [16] is also present during treatment.

Although screening enables identification of psychological distress and supportive care needs, its effectiveness depends on the availability of services capable of responding to identified concerns. When appropriate supportive care pathways are not available within oncology services, screening may function primarily as a method for documenting distress rather than facilitating access to meaningful treatment. The clinical value of distress screening therefore depends on the alignment between screening processes, referral options, and available supportive care resources within oncology settings. The disconnect observed in this study suggests there are opportunities to improve the translation of distress screening into clinical benefit for oncology patients. Taken together, these findings suggest that distress screening should be considered as one component of a broader system of psychological care, where identification of need must be accompanied by accessible and integrated referral pathways to link patients with appropriate support.

### 4.1 Clinical Implications

These findings highlight a missed opportunity to enhance patient care during treatment. Refinement of referral following screening and improvement of access to internal hospital services (particularly mental health services) may provide direct opportunity to better address expressed concerns and distress in this population. Integrating mental health professionals such as psychologists within oncology teams could enhance access to appropriate psychological support and reduce reliance on external services, which may be less accessible or disconnected from the patient's cancer care team. While external services remain important, internal referrals and service utilisation may better support effective multidisciplinary oncology care and would likely have a greater impact on patient experience.

### 4.2 Study Limitations

This study assessed referral following screening at a single institution, making the findings reflective of local resourcing and referral policies. The availability of local mental health services, institutional priorities, and hospital funding likely influenced results. This study is unable to determine whether patients were effectively connected with the services they were referred to and is only reflective of what referrals were made by hospital staff. Additionally, the small number of referrals to psycho‐oncology (*n* = 5) limited the ability to meaningfully assess factors associated with referral to specialist psychological services. These findings should therefore be interpreted cautiously and should not be interpreted as evidence that distress was unrelated to psycho‐oncology referral. It is also unknown from the available data the proportion of referrals which resulted in mitigation of distress and improved management of concerns raised during distress screening. While the focus of this study was on documenting referrals following screening rather than impact of referral, the inability to track outcomes of referral and resultant impact on patient distress from pre‐existing medical record data is a limitation in the screening process itself.

## 5 Conclusion

Psychological distress is common among patients receiving outpatient oncology care. However, the referrals following screening may not adequately address these concerns, and patients are frequently directed to external services disconnected from the oncology care team. To ensure that distress screening efforts lead to actionable interventions, hospital resourcing should be aligned with the psychological needs of patients, ultimately supporting an integrated, multidisciplinary approach to oncology care.

## Author Contributions

**E. Matthews:** conceptualization, data curation, investigation, formal analysis, methodology, project administration, writing – original draft. **K. Webber:** conceptualization, validation, writing – review and editing. **C. Parker:** conceptualization, validation, writing – review and editing. **C. Feola:** investigation, formal analysis, writing – review and editing. **T. Gencarelli:** investigation, formal analysis, writing – review and editing. **P. Koe‐Leong:** investigation, formal analysis, writing – review and editing. **J. F. Wiley:** conceptualization, investigation, formal analysis, methodology, validation, writing – review and editing.

## Conflicts of Interest

The authors declare no conflicts of interest.

## Supporting information


Supporting Information S1

## Data Availability

The data that support the findings of this study are available from the corresponding author upon reasonable request.
