# Supplementary material for: Identifying and Addressing Distress in Cancer Care: A Study of Screening Outcomes and Referrals in Australian Oncology Outpatients
Source: Psychooncology. 2026 Aug 20;35(8):e70584. doi: 10.1002/pon.70584 (PMC13492088; doi:10.1002/pon.70584)
Supplement: Supplementary file 1 [file PON-35-e70584-s001.docx]

Supplementary Materials

**Supplementary Table 1**

*Characteristics of the study population who declined screening*

| Age (years), *M (SD)* | 63.79 (13.24) |
| --- | --- |
| Age, *N (%)* |  |
| Adults (Age < 65 years) | 40 (48.8) |
| Older Adults (age ≥ 65 years) | 42 (51.2) |
| Language, *N (%)* |  |
| English | 55 (67.1) |
| Language other than English | 27 (32.9) |
| Sex, *N (%)* |  |
| Female | 57 (69.5) |
| Male | 25 (30.5) |
| Tumour Stream, *N (%)* |  |
| Brain | 0 (0.0) |
| Breast | 22 (26.8) |
| Genitourinary | 6 (7.3) |
| Gynaecological | 12 (14.6) |
| Head and Neck | 7 (8.5) |
| Lower Gastrointestinal | 8 (9.8) |
| Lung | 11 (13.4) |
| Skin | 1 (1.2) |
| Unknown Primary | 0 (0.0) |
| Upper Gastrointestinal | 15 (18.3) |
| Treatment Intent, *N (%)* |  |
| Curative | 50 (61.0) |
| Palliative | 32 (39.0) |
| Undetermined *^b^* | 0 (0.0) |

**Supplementary Table 2**

*Overall Prevalence of all Problem List Items Among Completed Screens*

| **Problem List item** | **Total number of screens that endorsed item** | **% of all screens endorsing item** |
| --- | --- | --- |
| Worry or anxiety | 363 | 47.27 |
| Fatigue | 305 | 39.71 |
| Pain | 292 | 38.02 |
| Sleep | 281 | 36.59 |
| Sadness or depression | 190 | 24.74 |
| Loss or changes of _physical abilities | 177 | 23.05 |
| Fear | 164 | 21.35 |
| Loss of interest or  _enjoyment | 149 | 19.40 |
| Changes in eating | 143 | 18.62 |
| Taking care of myself | 132 | 17.19 |
| Memory or concentration | 121 | 15.76 |
| Finances | 121 | 15.76 |
| Treatment decisions | 103 | 13.41 |
| Feelings of worthlessness  _or being a burden | 101 | 13.15 |
| Changes in appearance | 96 | 12.50 |
| Taking care of others | 88 | 11.46 |
| Work | 83 | 10.81 |
| Transportation | 81 | 10.55 |
| Anger | 72 | 9.38 |
| Relationship with spouse  _or partner | 56 | 7.29 |
| Loneliness | 55 | 7.16 |
| Grief or loss | 54 | 7.03 |
| Housing | 54 | 7.03 |
| Tobacco Use | 47 | 6.12 |
| Relationship with children | 46 | 5.99 |
| Relationship with family | 44 | 5.73 |
| Communication with  _health professionals | 41 | 5.34 |
| Death, dying or afterlife | 37 | 4.82 |
| Relationship with friends _or coworkers | 35 | 4.56 |
| Sexual Health | 31 | 4.04 |
| Having enough food | 27 | 3.52 |
| Access to medicine | 25 | 3.26 |
| Sense of meaning or  _purpose | 21 | 2.73 |
| Insurance | 20 | 2.60 |
| Conflict between beliefs _and cancer treatment | 20 | 2.60 |
| Substance Use | 13 | 1.69 |
| School | 12 | 1.56 |
| Child care | 8 | 1.04 |
| Ability to have children | 7 | 0.91 |
| Changes in faith or beliefs | 7 | 0.91 |
| Ritual or dietary needs | 5 | 0.65 |
| Relationships with the _sacred | 3 | 0.39 |
| Other | 0 | 0.0 |

**Supplementary Table 3**

*Number of Referrals Made Per Screening*

| **Number of referrals made** | **N screens** | **% of screened sample** |
| --- | --- | --- |
| 0 | 493 | 64.2 |
| 1 | 188 | 24.5 |
| 2 | 62 | 8.1 |
| 3 | 18 | 2.3 |
| 4 | 7 | 0.9 |

**Supplementary Table 4**

*Referrals Made by Demographic Variables*

|  | **Total number of screenings result in referrals** | **Total screens completed regardless of referral** | **% of screenings resulting in referral** |
| --- | --- | --- | --- |
| Sex | | | |
| Female | 138 | 423 | 32.6 |
| Male | 137 | 345 | 39.7 |
| Age | | | |
| Adults (Age < 65) | 139 | 390 | 34.9 |
| Older Adults (age ≥ 65) | 139 | 378 | 36.7 |
| Language | | | |
| English | 201 | 598 | 33.6 |
| Languages other than English | 74 | 170 | 43.5 |

**Treatment intent = N/A: treatment had not been determined at time of screening*
